# Thrombin and the *Coag-Inflammatory Nexus* in Neurotrauma, ALS, and Other Neurodegenerative Disorders

**DOI:** 10.3389/fneur.2019.00059

**Published:** 2019-02-05

**Authors:** Barry W. Festoff, Bruce A. Citron

**Affiliations:** ^1^pHLOGISTIX LLC, Fairway, KS, United States; ^2^Department of Neurology, University of Kansas Medical Center, Kansas City, KS, United States; ^3^Laboratory of Molecular Biology Research & Development, VA New Jersey Health Care System, East Orange, NJ, United States; ^4^Department of Pharmacology, Physiology & Neuroscience, Rutgers New Jersey Medical School, Newark, NJ, United States

**Keywords:** thrombin, thrombomodulin, PAR1, DAMPs, HMGB1, blood brain barrier, ALS, neurodegeneration

## Abstract

This review details our current understanding of thrombin signaling in neurodegeneration, with a focus on amyotrophic lateral sclerosis (ALS, Lou Gehrig's disease) as well as future directions to be pursued. The key factors are multifunctional and involved in regulatory pathways, namely innate immune and the coagulation cascade activation, that are essential for normal nervous system function and health. These two major host defense systems have a long history in evolution and include elements and regulators of the coagulation pathway that have significant impacts on both the peripheral and central nervous system in health and disease. The clotting cascade responds to a variety of insults to the CNS including injury and infection. The blood brain barrier is affected by these responses and its compromise also contributes to these detrimental effects. Important molecules in signaling that contribute to or protect against neurodegeneration include thrombin, thrombomodulin (TM), protease activated receptor 1 (PAR1), damage associated molecular patterns (DAMPs), such as high mobility group box protein 1 (HMGB1) and those released from mitochondria (mtDAMPs). Each of these molecules are entangled in choices dependent upon specific signaling pathways in play. For example, the particular cleavage of PAR1 by thrombin vs. activated protein C (APC) will have downstream effects through coupled factors to result in toxicity or neuroprotection. Furthermore, numerous interactions influence these choices such as the interplay between HMGB1, thrombin, and TM. Our hope is that improved understanding of the ways that components of the coagulation cascade affect innate immune inflammatory responses and influence the course of neurodegeneration, especially after injury, will lead to effective therapeutic approaches for ALS, traumatic brain injury, and other neurodegenerative disorders.

## Introduction

**Figure 1:**
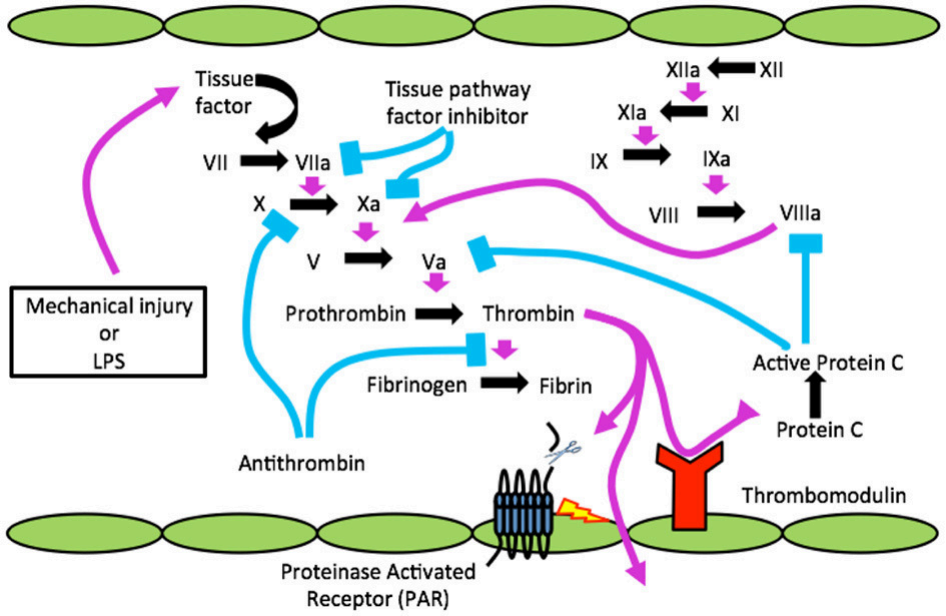
The clotting cascade, TBI, infection (LPS) and the blood brain barrier. Cartoon shows injury or infection, lipopolysaccharide (LPS) releasing tissue factor (TF) to activate clotting resulting in active α-thrombin, which cleaves a receptor(s) known as PAR on endothelial cells (ECs) to disrupt BBB. Through disrupted BBB thrombin gains access to CNS and can cleave to activate PARs on microglia/astrocytes for neuroinflammation and neurons to form neurofibrillary tangles (NFTs).

In humans, the coagulation system or cascade was conceptualized over the past five to six decades to consist of five serine proteases (factor VII, FVII; factor IX, FIX; factor X, FX; protein C, PC and prothrombin, PT) that act with five cofactors (tissue factor, TF; factor V, FV; factor VIII, FVIII; thrombomodulin, TM; and protein S, PS) to control the generation of fibrin, which is subsequently cross-linked by Factor XIII (FXIII), a transglutaminase (1). This system is essentially conserved throughout mammalian species (schematically shown in Figure 1), but the system's endpoint, hemostasis, has been around for 450 million years. Hemostasis consists of three activities that are closely regulated; vasoconstriction, platelet aggregation, and clotting factor activation. Two different pathways, the *intrinsic* (contact) and *extrinsic* (TF), exist to activate clotting and the principal difference is the role of TF in the extrinsic pathway, which works very rapidly. With blood vessel damage, *inactive FVII* comes in contact with TF, a protein on the endothelial cell (EC), and activates it to a protease (2). Activated Factor VII then proteolytically activates *FX* that then binds *activated FV* to form *prothrombinase*. So, recapping, TF release is very rapid and generated by damaged blood vessels and surrounding tissues, which is especially high in brain, and initiates the extrinsic pathway.

Since endothelial cell damage is the principal mechanism for clotting factor activation via TF generation it invariably occurs with systemic microbial infection, as in sepsis, where the innate immune system is activated (2). Indicating sepsis in Figure 1 is bacterial LPS (lipopolysaccharide or endotoxin). It was subsequently found that trauma, a sterile injury, also produces TF-generated coagulation (Figure 1) (3).

The clotting system is involved in host defense, and arose with and is linked to innate immunity or inflammation at very early evolutionary stages. TF is the key actor and common generator providing the critical *nexus* between these two major host defense systems (4). TF belongs to the cytokine receptor superfamily and is a type I integral membrane glycoprotein (5). Thrombin, the ultimate serine protease in the cascade, is the key downstream product of TF-initiated coagulation. Not only does it play a central role in hemostasis but more recent studies have revealed its fundamental and intense proinflammatory effects (6). These latter attributes of thrombin, just as its role in causing platelet aggregation, were subsequently ascribed to its non-coagulation actions as a ligand for cell-surface receptors, now known as protease-activated receptors (PARs) (7–9).

Although these thrombin-mediated, PAR-activated cellular effects involve thrombin's roles in cell proliferation and modulation, cytoprotection and apoptosis, its role as a proinflammatory mediator is key that further brings together coagulation and inflammation—the *coag-inflamm nexus*. Furthermore, it incorporates innate immune pathways such as toll-like receptors (TLRs) and complement, exosomes/microparticles (MPs) into this nexus. With cellular activation thrombin also recruits other systems to provide a balance for this coag-inflammatory pressure, and this includes the *protein C (PC)–thrombomodulin (TM)* natural anticoagulant/anti-inflammatory machinery along with activation and monitoring of the fibrinolytic system.

In the 1980's a few studies began to explore the direct effects of thrombin on cultured neural cells (10–13). Those initial reports ushered in a number of successive studies of thrombin, the coagulation and fibrinolytic cascades, TM, PARs in the CNS that continues to the present time. More recent efforts at translation of tissue culture and animal studies to neurologic diseases are now chronicled in other reports in this Frontiers in Neurology collection.

## Amyotrophic Lateral Sclerosis (ALS) and Neurodegenerative Disorders

Amyotrophic lateral sclerosis (ALS) is a neurodegenerative disorder exemplified clinically by muscle weakness and wasting and neuropathologically by degeneration of upper and lower motor neurons in the spinal cord, brain and brainstem (14–16). More recent evidence indicates that a number of *endophenotypes* exist for ALS beyond what was considered 30–50 years ago: the four *classic* motor neuron disorders. These are: classical ALS (upper and lower motor neuron and bulbar involvement), progressive muscular atrophy (PMA; only lower motor neuron), progressive bulbar palsy (PBP; brainstem with little if any extremity features) and primary lateral sclerosis (PLS; only upper motor) if it is actually part of the spectrum. As a distinct *nosologic* disorder ALS has been known in the medical literature since Charcot first described it 150 years ago in the late nineteenth century (17).

It is a fatal and currently enigmatic disease with death usually resulting from the inexorable progression of diaphragmatic and intercostal muscle weakness ultimately causing paralysis and respiratory failure typically within 5 years of diagnosis. The incidence of ALS has changed only slightly since the 1970 s and is ~1.5–3 per 100,000 in Western Europe and North America with little variation. It is overwhelmingly a sporadic disease (sALS), but genetic variants exist (fALS) accounting for no more than 10% of all cases (see below), although newer information may be changing this. ALS has an estimated lifetime risk of 1 in 400, is an adult-onset illness that is rare before the age of 40 years increasing exponentially with age. There are no known treatments that impact progression of the disease. Until 2017, the last Food and Drug Administration (FDA) approved drug was Riluzole™, licensed in 1996 and that only extended survival of ALS patients 3 months. In May 2017 the FDA approved edaravone (Radicava™) to treat ALS patients based on a 2nd Phase 3 study after the first was negative (18). As the authors wrote: the drug “….showed efficacy in a small subset of people with ALS who met criteria identified in *post-hoc* analysis of a previous phase 3 study, showing a significantly smaller decline of ALSFRS-R score compared with placebo.”

As discussed in detail below, our laboratory at the Kansas City VA Medical Center began studies of the coagulation system in ALS in the 1980's (see Table 1).

**Table 1:** Partial history of thrombin signaling in CNS and PNS: Key processes involving neural and neuromuscular health and the thrombin signaling pathway.

**Event**	**References**
Release of acetylcholinesterase (AChE) in ALS and denervation of muscle	(19–26)
Denervation and plasminogen activators	(27–38)
Thrombin, protease nexin I, PAR1, and synapse formation/elimination	(39–52)
Thrombospondin and ALS	(53–59)
Coagulation, astrocytes, BBB and neuroinflammation	(60–64)

## Genetics and ALS: familial ALS (fALS)

Although it was considered a sporadic illness beginning in the 1990 s interest in the ~5–10% of ALS cases that had family history began. Identification of mutations in the superoxide dismutase 1 gene (*SOD1*) was reported in 1992 (65, 66). Over the next 25 years remarkable progress in our understanding of SOD1 and fALS has occurred (67–73).

Even amongst otherwise sALS cases about 1–3% possess missense mutations in SOD1 (74) and even more, about 5–10% of sporadic ALS cases are caused by intronic expansions in *C9orf72*, the open reading frame (ORF 72) on chromosome 9 (75–77). This indicates that 1 in 20 cases of sALS and about 40% of fALS are due to *C9orf72* hexanucleotide repeats.

With SOD1 and *C9orf72* more than 20 mutated genes have now been found to be specifically associated with fALS (78) that include *TARDBP* (79–81) and *FUS* (82, 83), the *fused in sarcoma* gene on chromosome 16p11.2, that is involved with RNA processing, which together with *SOD1* and *C9orf72*, are the four most common genes involved in causing ALS clinically. TARDP encodes a protein, TAR DNA binding protein (TDP-43) that accumulates in most sALS motor neurons but not SOD1 fALS neurons (84). These genes have been numbered now as ALS1-ALS22, along with FTDALS1, FTDALS 2, FTDALS 3, and FTDALS 4 (78). Genome-wide association studies (GWAS) may be changing the role of genetics in ALS including what we now consider sALS (85–87).

The changing viewpoint results from studies of the relatively uncommon genetic cases of this enigmatic and fatal neurodegenerative disorder that have revealed some fundamental clues that might uncover novel therapeutic targets. Amongst these are more recently identified *endophenotypes* beyond the classical motor sub-types. Endophenotypes are inherited traits identified using clinical or laboratory measures including electroencephalographic or electromyographic abnormalities, neurocognitive deficiencies, and other modalities that identify impairment. Until recently they have been largely used in psychiatric and psychopathology-related research. Originally conceived by Gottesman and Shields (88), they were proposed to appear not only in patients but also in their unaffected relatives. The presumption of endophenotypes is that they are more proximate to gene action than the clinical diagnoses (89, 90). In neurodegenerative diseases such as Parkinson's (PD) and Alzheimer's (AD) diseases, in addition to ALS, they might provide dual positives such as improving diagnoses and initiating therapy in preclinical stages (91–93).

### ALS Endophenotypes Beyond the Motor System

ALS is now recognized as a multi-system neurodegeneration rather than a disease limited to motor neurons (94–97). Although 40 years ago if a patient was clinically diagnosed with ALS but exhibited cognitive symptoms that patient was not considered to have classical ALS and typically was removed from consideration. In fact, in the original *El Escorial* criteria (98) and El Escorial revisited (99, 100), the presence of dementia essentially ruled out ALS as diagnosis. This action was taken despite the fact that descriptions of cognitive and behavioral symptoms resembling frontotemporal dementia (FTD) in otherwise typical ALS motor phenotypes date back to the 1880 s. The neurologic giant, Arnold Pick, whose name is eponymic for a subgroup of FTD known as Pick's disease, was aware that Charcot had considered that non-motor brain regions might also be involved in the neurodegeneration of what is now known as *Maladie de Charcot* or *la sclérose latérale amyotrophique (SLA)* by francophones.

One of the first descriptions of FTD associated with ALS in the modern era was provided by the late Canadian neurologist, Arthur Hudson (101), who also described mixed types of ALS with parkinsonism as well as with dementia and other clinical features, reminiscent of the *ALS-parkinsonism-dementia* complex of Guam (102, 103). Since then increasing interest in FTD-like symptoms in ALS patients appeared and it is now thought that about 10% of patients with one of the four classic motor-neuron disorders: classical ALS, PMA, PBP, and PLS, have cognitive features.

Subsequent reviews of the ALS/FTD complex have appeared (104, 105) that now also include associations with *C9orf72* expansions (77, 96, 106, 107). In fact, the seminal discovery of a GGGGCC hexanucleotide repeat expansion (HRE) within the chromosome 9 ORF 72 (75–77, 108), has been established as cause for the most common form of ALS/FTD (107).

Given this common cause of sALS with FTD, 10% of sALS and an additional 10% of FTD, the next question, given that it has taken more than 25 years with SOD1 mutations, is just how cytotoxicity occurs with the GGGGCC (G4C2) HRE within the C9ORF72 gene? Several recent studies have shed light on this: it has been reported that HRE RNA forms hairpin and G-quadruplex structures that bind and sequester RNA-binding proteins (RBPs). The GGGGCC are translated into specific dipeptide-repeat (DPR) proteins, and these form toxic aggregates, particularly the arginine-rich dipeptides, specifically proline-arginine (PR), that possess potent neurotoxicity forming aggregates in nuclei and nucleoli, and stress granule formation, with likely effects on translation (109). These authors used inducible pluripotent stem cells (iPSCs) to differentiate into human motor neurons (iMNs), including those from ALS patients carrying the repeat-expanded *C9ORF72*. These studies revealed that C9ORF72 in the ALS patients was *haploinsufficient*. Thus, the ALS/FTD gene had only one functional copy, causing a loss-of-function mutation. Using blood cells from healthy individuals they used gene-editing techniques to delete the *C9ORF72* or from ALS patients with the abnormal gene. They found that C9ORF72 cooperated with endosomes, was involved in vesicle trafficking and formation of lysosomes in motor neurons. When they repeated the nucleotide expansion this reduced *C9ORF72* expression, and with this process, neurodegeneration was triggered via both gain- and loss-of-function mechanisms. The former produced a buildup of glutamate receptors, causing excitotoxicity, while the latter weakened neurotoxic dipeptide repeat proteins clearance derived from the repeat expansion. This cooperative action led to neurodegeneration. These and other researchers have begun using the gene-editing tool, CRISPR, specifically the CRISPR–Cas9 system, to perform genome-wide gene-knockout screens similar to studies in cancer (110).

Frontototemporal lobar degeneration (FTLD) is the 2nd most common cause of dementia in elderly (over age 65) individuals and is actually a broad spectrum of neurological disorders. FTD is a variant of FTLD and from GWAS studies now appears to share a number of genetic as well as clinical and neuropathological features. In a recent GWAS study of more than 120,000 neurodegenerative diseases and controls unique genetic overlap between ALS and FTD spectrum diseases was found (111). Of interest, the H1 haplotype of the tau protein gene (MAPT) appeared to confer risk for ALS, as did *BNIP1*, a mitophagy-associated, proapoptotic protein.

If an *endophenotype strategy* in ALS should be implemented, as has since been undertaken in several neurodegenerative studies, it will depend both on quality and properties of a specific trait. It will be necessary to critically evaluate the trait(s) to determine if it truly can capture pre-diagnosis features of ALS/FTD. However, no consistency has yet appeared for endophenotypes or intermediate traits or even biomarkers, but some encouraging signs have appeared (112, 113). When such validated intermediate traits or biomarkers are considered, it will be necessary to forgo requiring that they be absolutely specific for ALS or FTD. Consequently, application of endophenotypes to future analyses of ALS and FTD seems more than justified.

With consideration of the ALS spectrum as a *non-cell autonomous* condition (114, 115), it brought to the picture the evidence that glial cells, including astrocytes, oligodendrocytes, and even microglia play important roles in the pathogenesis of ALS (116–118). Prior to the last decade it was widely assumed that motoneuronal cell death proceeded by cell autonomous mechanisms. However, information gained initially from using SOD1 transgenic mice and subsequently with other genetic models, the non-cell autonomous position evolved. In terms of *SOD1* more than 170 different mutations have been shown to cause fALS. When SOD1 mutations were expressed only in neurons neurodegeneration did not occur in the mice (119). But just how these mutations in SOD1 result in cytotoxicity is still unclear, despite more than 25 years of study. In fact, no consensus has emerged as to the principal mechanism for neurotoxicity or even how cells might protect themselves from it. Cleveland and colleagues proposed that ALS was just the tip of the iceberg and that non-cell autonomy will be shown to be the mode in other neurodegenerative diseases (114, 115, 120, 121).

Along these lines the multi-faceted roles of astrocytes have now become prominent for investigation in ALS (115, 118, 122–124). Discussed in more detailed below, reactive *astrocytosis* also known as astrogliosis, is a classic glial response to CNS injury and scar-forming reactive astrocytes are usually viewed as detrimental to clinical outcome (125), but not always (126). Astrocytes are also hallmarks of neurodegeneration (127) and using the ME7 prion mouse model Cunningham and colleagues showed that neurodegeneration primed astrocytes to produce exaggerated chemokine responses when stimulated with acute proinflammatory cytokines (128). The usual neuropathologic means to characterize reactive astrocytes is by using antibodies to the intermediate filament glial fibrillary acidic protein (GFAP). However, all phenotypes of astrocytes including reactive astrocytes and scar-forming astrocytes strongly express GFAP. In fact, being able to modulate extent and phenotypes of reactive astrocyte function (129) is potentially attractive as novel targets to enhance the functional outcomes after spinal cord injury (SCI) (130) or in ALS and other neurodegenerative diseases might be revealed (116, 131).

## Connecting Dots To Neurodegeneration: Neuroinflammation, Coagulation, BBB

### Inflammatory, and Innate Immune Aspects of ALS

Reviewing numerous studies of the past two decades has divulged previously held concepts that upper and lower motor neurons were the focus of ALS disease burden have now been replaced by non-cell autonomous mechanisms. Such non-cell autonomous mechanisms, particularly neuroinflammation, may not only contribute to the disease process but may initiate it, as detailed below.

Based on several lines of evidence within the last 20 years both sALS and fALS have had numerous proinflammatory markers associated with them (132–135). More than two decades ago, Appel et al. emphasized potential autoimmunity in ALS (136–138), and several different approaches revealed that immunoglobulin G (IgG) from ALS patients' sera caused toxicity in cultured motor neurons and in mouse models (138–141), with activation of L-type Ca^2+^-channels.

As exception proving the rule or standing in apparent contradiction, since it was present in the context of immunodeficiency, was our earlier report documenting ALS in a young homosexual male patient in whom HTLV-III (subsequently re-named HIV) was isolated (142). This initial observation was later confirmed in more recent accounts (143–147), suggesting that ALS, if truly autoimmune, may also be associated with immune deficiency disorders such as AIDs.

By definition, neuroinflammation is inflammation of nervous tissue and is characterized by proliferation and activation of glial cells, primarily microglia, and astrocytes, as well as transmigration of circulating immune cells, including polymorphonuclear neutrophils (PMNs), monocyte/macrophages, and T lymphocytes (T-cells) into the parenchyma across the blood-brain barrier (BBB) (148–152). In addition to these cellular characteristics, neuroinflammation includes humoral features such as proinflammatory cytokine and chemokine overproduction, along with their respective receptors (151). Of relevance here are the numerous reports of neuroinflammation in both sALS and fALS including its appearance in pre-symptomatic phases in transgenic mice. However, confusion has developed from these data since both deleterious and beneficial effects have been found especially when focusing on motor neuron survival and also depending on what disease stage was examined (153, 154).

#### Microglia

The understanding of these complex interactions largely centers on microglia, considered the brain's resident macrophages, and their dual roles or *Janus faces*, in neurodegeneration in general and ALS in particular (107, 153). In essence, although microglial phenotypes were classified as either M1 (“classically activated”) or M2 (“alternatively activated”), similar to circulating macrophages, phenotypic diversity of microglia is actually a spectrum (155). M1 microglia could be considered more proinflammatory while M2 more anti-inflammatory, possibly viewed as “deactivated” after phagocytosis of apoptotic cells. Clearly, a therapeutic strategy in ALS or in AD or PD for that matter, might be to selectively modulate microglial phenotypes, such as inhibiting or blocking M1 or enhancing M2. That may be too simple, although it is a strategy worth evaluating. However, this should not be done with pre-clinical animal models due to known differences in inflammatory responses compared to humans, but in human iPSC *ex vivo* models that incorporate elements of the blood-spinal cord barrier (BSCB)BBB/NVU along with neurons (156, 157).

#### Astrocytes

Of the several types of glial cells in the CNS astrocytes are the most abundant. Classically considered “supportive” cells for neurons astrocytes have recently been shown to be critical in regulating CNS immunity, but exactly how they do this is largely unknown. Astrocytes have been shown to be regionally diverse within the brain and in the spinal cord. Regions where astrocytes may be involved in regulating CNS immunity are at their “end-feet” localized to where they are contiguous with ECs of the BBB and neurovascular unit (NVU) as well as perivascular end-feet that form the *glia limitans*. All astrocytes are ramified and have processes that terminate on basal lamina impacting the perivascular compartment with their end-feet (127).

Of interest, one molecule highly concentrated in astrocytic end-feet is the gap junction protein, connexin 43 (Cx43) (158). Cx43 may have roles in the non-cell autonomous pathogenesis of sALS, implicating toxic mitochondria transferring from astrocytes to motor neurons at the BSCB (159, 160), as detailed below.

By analogy to macrophages, the M1/M2 macrophage and microglial nomenclature (161), although with caveats for its potential simplicity researchers have also applied these to reactive astrocytes (125, 127) into A1 and A2 sub-classes (162), whether caused by neuroinflammation or ischemia, respectively. As with microglial M1 and M2 sub-classes the macrophage phenotypic literature clearly indicates that these circulating immune cells display more than two polarization states (155, 163, 164). Chronic neurodegeneration also produces changes in the secretory profile of astrocytes in terms of what cytokines and chemokines are produced (128).

As M1 macrophages were considered destructive, so, too, are A1 astrocytes. Conversely, since M2 were considered reparative and protective as a macrophage or microglial phenotype, so were the A2 astrocytes. Liddelow, in the late Ben Barres' group, further showed that A1 were induced by reactive microglia (165).

## Innate Immune Activation in ALS

Over the past two decades our thinking about the brain and spinal cord as being an immunologically privileged site has changed. It was previously thought that the CNS could not mount an immune response nor process antigens. More recent studies have reversed that indicating that immune surveillance does take place in the CNS, and glial cells of all types act as immune effector cells within the CNS (149, 166). We now know, for example that the primary function of the CNS innate immune system is to provide neuroprotection against invading pathogens. However, in addition to infectious agents it is also protective for injury stimuli, and by so doing maintains CNS homeostasis.

### Pattern Recognition Receptors, Pathogen-Associated Molecular Patterns in ALS

Knowledge of how membrane and intracellular receptors respond to pathogenic components dramatically increased with identification of pattern recognition receptors (PRRs) to identify pathogen-associated molecular patterns (PAMPs), the prototype for which is lipopolysaccharide (LPS) or endotoxin, from Gram-negative bacteria. The effect of LPS in the CNS is to cause *sickness behavior*, a coordinated set of adaptive behavioral changes to LPS and others (166–168) that includes: fever, anorexia, social withdrawal, lethargy, and decreased rapid-eye movement sleep (REMS). Major innate immune system PRRs such as the Toll-like receptors (TLRs) and the receptor for advanced glycosylation endproducts (RAGE) are expressed in the CNS. Most TLRs, now 15 members of the family, and RAGE, are expressed in all neural cells (167–174).

### Damage-Associated Molecular Patterns (DAMPs)

The *danger-damage theory* expressed in 1994 by Polly Matzinger (175–177) changed the concept of immunology from simply detecting self vs. non-self. Her thesis was that the immune system's driving force is the *need to recognize danger and prevent destruction*. This theory evolved with publication of the proceedings of the EMBO Workshop on Innate Danger Signals and HMGB1 that took place February 2006 in Milan, Italy (organized by M. Bianchi, K. Tracey, and U. Andersson) (178). In keeping with this concept a group of endogenous molecules that signaled damage or danger were developed and were referred to as *alarmins*, a sub-category of DAMPs. Subsequent studies indicated that the PRRs recognized and responded to DAMPs in essentially the same manner as their response to PAMPs (177, 179, 180) and that the CNS also participated (181). The comparison of PAMPs and DAMPs and list of both is shown in Table 2.

**Table 2:** PAMPs and DAMPs: danger/damage recognition systems extrinsic and intrinsic.

**Patterns**	**Characteristics**
Pathogen-associated molecular patterns (PAMPs)	Prototype is lipopolysaccharide (LPS; endotoxin)
	Activate several pattern recognition receptors (PRRs)
	PRRs include Toll-like (TLRs) and advanced glycation end-product (RAGE) receptors
	Highly Conserved among diverse species
	MAMP (microbe-associated molecular pattern) may be more accurate
Damage-associated molecular patterns (DAMPs)	Prototype is high mobility group box protein 1 (HMGB1)
	Non-histone DNA binding, nuclear protein
	Also present in mitochondria (mtDAMPs), like TFAM
	Activated late in sepsis, necrotic and dendritic cells
	Released with trauma, necrosis, including in the CNS

**Figure 2:**
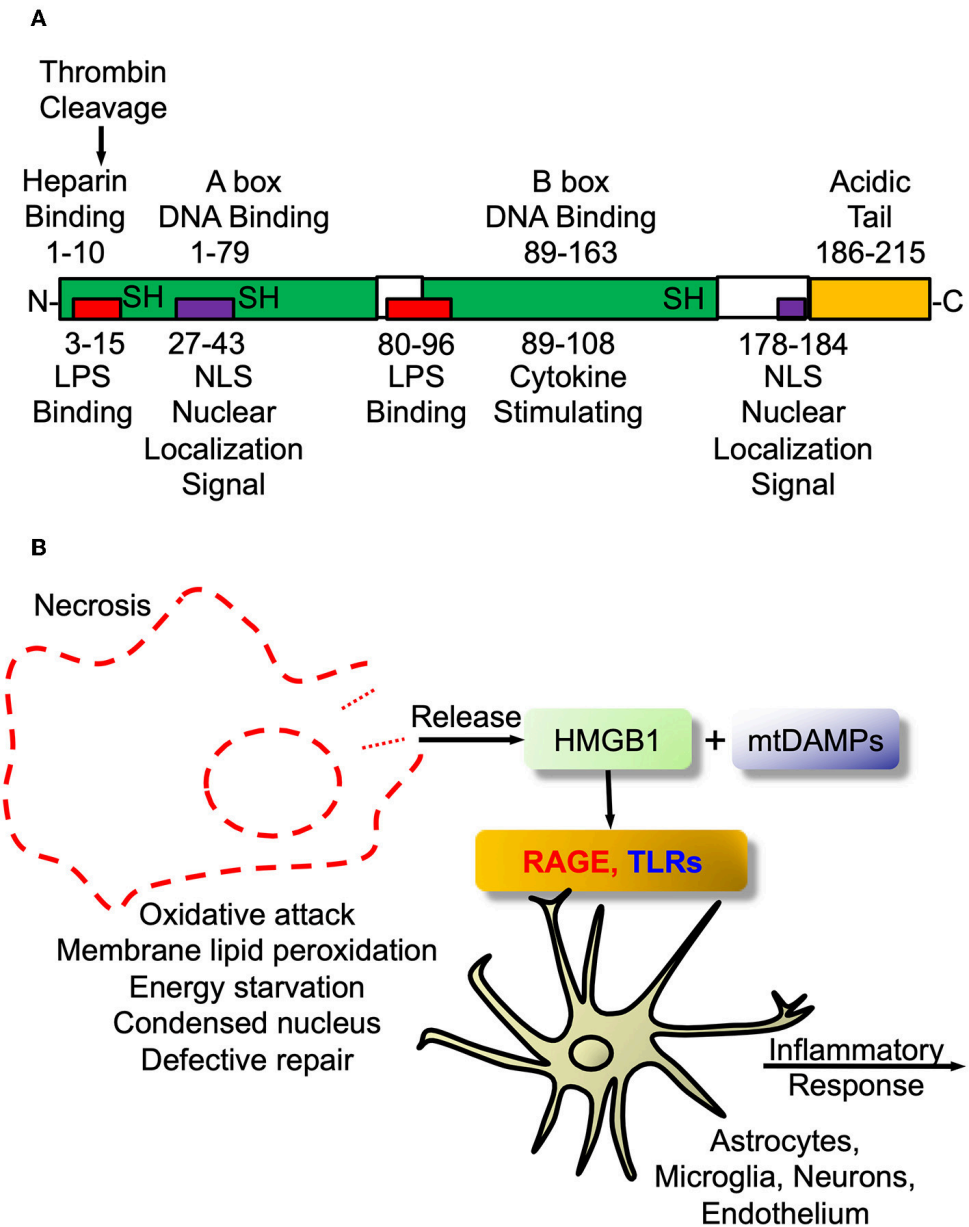
High mobility group box protein 1 **(**HMGB1). **(A)** HMGB1 Structure. Schematic of HMGB1's two binding motifs, A-box and B-box. Also showing critical cysteines that determine whether it is proinflammatory when outside cell or DNA binding when inside nucleus. In addition, the C-terminal acidic tail appears to confer protein stability and DNA bending *in vitro*. **(B)** HMGB1 signaling. The damage-associated molecular pattern (DAMP) HMGB1 is passively released from necrotic or exploding cells such as with infection or trauma. Within the CNS it engages pattern recognition receptors (PRRs) such as toll-like receptors (TLRs2/4) and receptor for advanced glycation end products (RAGE) to initiate proinflammatory signaling as part of innate immune activation.

In fact, as an example that science, certainly more in the pre-cloning era, was guilty of the *blind men describing the elephant* parable, Finnish workers had identified a protein that guided early neuroblasts to their final locations in developing mouse brain and called this protein, *amphoterin* (182, 183). Amphoterin, also called P30 protein, was subsequently found to be identical to HMGB1 (170), the prototypic alarmin/DAMP. The structure of the alarmin/DAMP HMGB1 is shown in Figure 2A and its known signaling in Figure 2B. The relationship between HMGB1 and thrombin is interesting. Both are prototypes of ancient host defense systems, inflammation and coagulation (60), but in addition, as shown in Figure 2B, thrombin can cleave HMGB1 at its –NH2 end and does so when bound to TM (184). Of interest, since then HMGB1 has been shown to be involved in a number of neuropathologic processes in the CNS and is also essential for brain development (181, 185).

As mentioned above PRRs, especially TLRs and RAGE, expressed by immune cells are also expressed by neural cells, particularly astrocytic and microglial, to mediate resident immune cell activation (169, 172). As described for AD and other neurodegenerative diseases (63, 148, 181, 186), DAMPs are probable candidates to partake in, and possibly initiate, ALS neurodegenerative activities. HMGB1 is over-expressed in SOD1 mutant mouse spinal cord and motor cortex and from patients with ALS (187). TLRs were also found to be over-expressed in ALS patients' spinal cords (188), as was RAGE, along with its proinflammatory ligands, including HMGB1, S100B and calgranulin (189). Furthermore, a number of groups have focused on levels of circulating soluble RAGE (sRAGE) in various diseases including diabetes mellitus, cardiovascular and neurodegenerative diseases (190, 191), recently including ALS (174, 189, 192). As opposed to sRAGE being “specific” for any of those diseases it is clear that it is implicated in their pathogenesis and contributes to our understanding of innate immunity in these conditions. Furthermore, it might be useful as therapeutic strategy in one or more of them. Additionally, sRAGE has been considered a “decoy receptor” to block the cellular membrane receptor to block RAGE-mediated signaling. In this regard, sRAGE is decreased in blood while increased in affected CNS in ALS and other neurodegenerative diseases (193).

## Mitochondria, ALS, and mtDAMPs

A key mechanism whereby motor neurons degenerate in ALS is by influence of dysfunctional mitochondria (194–196). As in PD and AD and other neurodegenerative diseases, studies in SOD1 transgenics as well as in sALS cells have been performed that show such mitochondrial defects, with an eye to novel therapeutics (197–199). Almost 20 years have elapsed since the close temporal relationship of the onset of motor neuronal degeneration with initiation of astrogliosis in the SOD1 mouse model was first identified (200). With further understanding of the non-cell autonomous, specifically astrocytic, aspect of ALS pathogenesis abnormalities in astrocyte mitochondria have been found (201–205). In particular, the demonstration that “positive” aspects of mitochondria can be shifted to neurons in transcelluar organelle transfer (159) indicates that negative or toxic aspects of astrocytic mitochondria might be transferred to motor neurons in sALS or fALS (206, 207), possibly via connexin 43 (159, 160).

**Figure 3:**
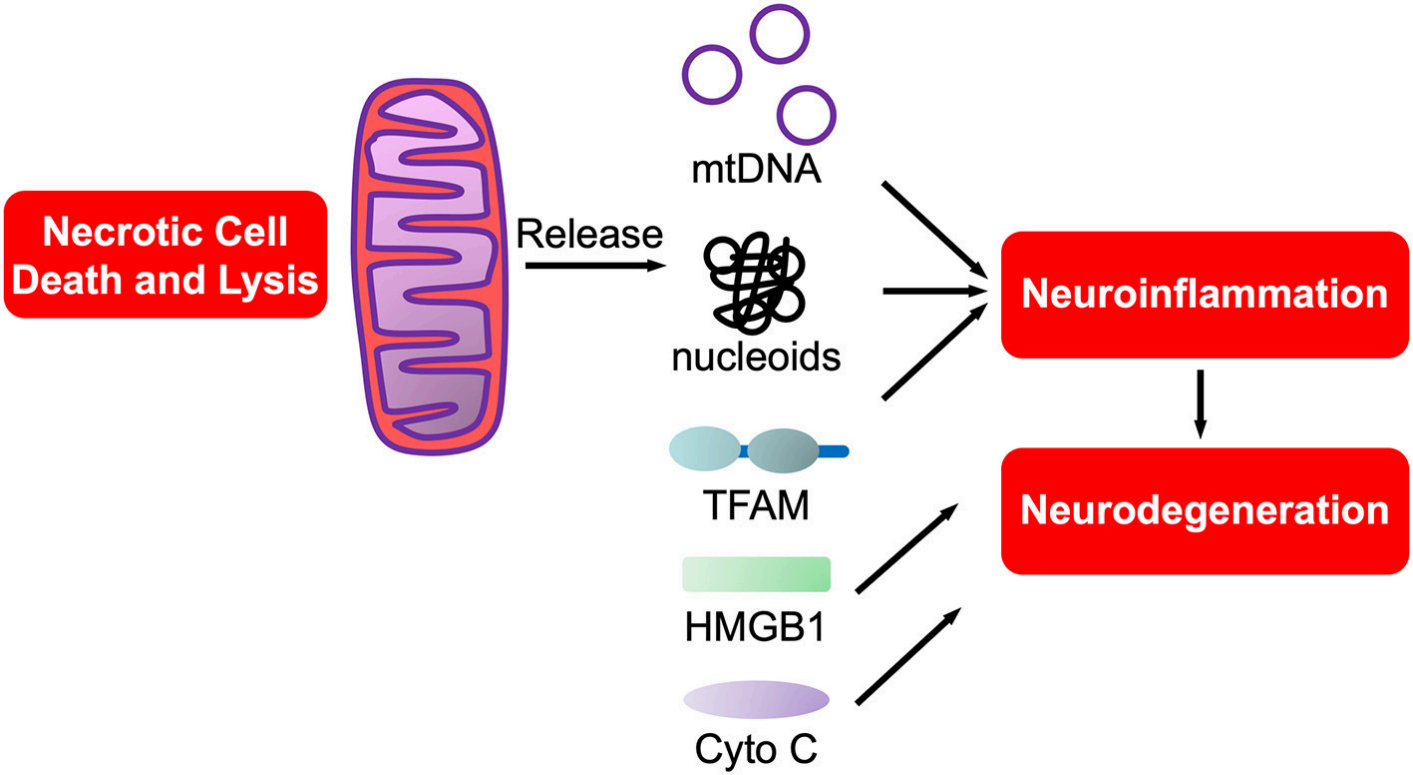
mtDAMPs. A sub-category of DAMPs, mtDAMPs are components of mitochondria released from necrotic or exploding cells. Comprised of mtDNA bound to mitochondrial proteins such as transcription factor of mitochondria A (TFAM), a homolog of HMGB1, in nucleoids. Also includes n-formyl peptides, cytochrome C and others (not shown).

Of interest, aligned with the “danger theory” is the *endosymbiotic* theory that mitochondria originated from protobacteria that entered into an endosymbiotic relationship with phagocytic, unicellular anaerobes at least a billion years ago (208), prior to the accumulation of oxygen in the atmosphere. Mitochondrial DAMPs (mtDAMPs), are protein DAMPs, coded for by mitochondrial or nuclear genes, that when released from mitochondria are potently proinflammatory (209). Most mtDAMPs are encoded by nuclear genes that after transcription translocate from nuclei to mitochondria. These mtDAMPs are then released into the circulation with infection (sepsis), trauma and/or systemic inflammatory response syndrome (SIRS). In support of this being relevant in the CNS we showed that mtDNA, a nucleic acid mtDAMP, was potently proinflammatory for neurons and microglia (210). Of interest, PCR-amplified purified mtDNA was not proinflammatory, rather only brain isolated mtDNA in the form of nucleoids bound to transcription factor of mitochondria A (TFAM), itself a mtDAMP (211, 212), was proinflammatory (211, 212). Such studies support the prediction that mitochondrial dysfunction in neurodegeneration, and neurotrauma, is tightly linked to neuroinflammation (151, 213), especially with mtDAMP involvement in neurodegeneration (214). The potential roles of mtDAMPs in neurodegeneration are shown in Figure 3.

## Blood-brain Barrier (BBB)

Just as the CNS responds to PAMPs like LPS so does it respond to DAMPs, both initiating proinflammatory signaling and for the bridging or disruption of the BBB (63, 64). The history of a perceived association between ALS and BBB dysfunction actually dates back to the 1940 s when Robert Aird began a 40 year involvement with the BBB in neurologic diseases (215–219). However, it took several more decades before technology caught up with the concept that ALS was associated with BBB or blood-spinal cord barrier (BSCB) dysfunction, perhaps even at the onset of the disorder (220–231).

Evans blue extravasation from capillaries into spinal cord parenchyma was found in early symptomatic SOD1 transgenic (G93A) mice but it was uncertain whether BBB/BSCB disruption was cause or effect of motor neuron degeneration (220, 221, 223, 232, 233). More evidence suggestive of causative influences have since appeared with further studies of SOD1 transgenic mice with an eye toward therapy as well (233–235). This situation is fundamentally the same for neuroinflammation in ALS—that is, is it cause or effect? The findings that the *C9orf72* expansions are also associated with myeloid cell abnormalities and early BBB dysfunction supports the role of these processes in pathogenesis (107, 236). The concept of the BBB being both *target* of circulating coag-inflammatory molecules as well as the *source* of pro-(neuro)inflammatory mediators is shown by Festoff et al. (63).

## Neurotrauma and ALS

The progression of neurodegenerative disease following neurotrauma is both anecdotal and supported by epidemiologic statistics. Dementia, including AD, and microvascular dementia (mVAD), is now considered to have increased risk following traumatic brain injury (TBI), while the specific mechanistic details are still under study. Similarly, ALS occurs at an increased risk following TBI, more so, in fact than after typical SCI. Numerous mechanisms have been suggested for this association of neurotrauma and neurodegeneration including increased interest for almost 30 years in the nexus of inflammation, BBB disruption and coagulation.

Early reviews of mechanical and other forms of spinal and CNS injury associated with the development of ALS have appeared, some positive while others were negative (237, 238). Case-control studies, however, are few and those, such as that published from Olmsted County, Minnesota by Kurland and colleagues were not supportive (239). However, more recent larger population-based case control investigations, such as the Danish study, have shown an association especially with trauma at an early age (240, 241). Even broader studies such as the European EURALS consortium study (242) are giving credence to a role for trauma in ALS pathogenesis. This large study showed that more than 2 head injuries was associated with >3-fold increased risk of ALS. Although the site of injury was not important the risk was only ~2-fold when trauma occurred between 35 and 54 years of age. Certainly the age at first trauma might help to explain discrepancies in results of past studies of trauma and ALS.

In addition, studies of chronic traumatic encephalopathy (CTE) with repeated mild traumatic brain injury (mTBI) or concussions (243–245), indicate that there may be methods to identify, monitor and treat and/or prevent neurodegenerative disease development in the context of neurotrauma. With more thorough investigation into CTE and former professional athletes, an increased incidence of clinical ALS diagnosis has been reported. A recent study of CTE and CTE associated with ALS (CTE-ALS) confirmed that molecular changes co-existed pathologically. Specifically, these were phosphorylation of tau at threonine 175 (Thr175) and at Thr231 along with GSK3β were found in these tauopathies (246). Furthermore, similar findings were present in rats subjected to moderate TBI in a controlled cortical impact (CCI) model (246). These findings suggest that comparable underlying molecular mechanisms for abnormal tau phosphorylation associated with CTE neuropathologic aspects may be mimicked in a rat moderate TBI model. However, they do not provide evidence for a neurotraumatic basis for sALS.

What neurotrauma does tell us for ALS is that there is a distinct relationship between trauma, BBB disruption and neuroinflammation (247–250), all potential contributing pathogenetic factors in ALS. From the BBB disruption perspective, mechanisms involved with TBI include impact-induced shear force stress that causes initial vascular injury followed by escape of proteins along with extravasation from brain to blood as well macromolecule leakage and cell transmigration from blood to brain.

## Coagulation Aspects of ALS and Other Neurodegenerative Diseases

Beginning in the 1980s through the 2000's our laboratory focused its attention on thrombin, the ultimate serine protease in the coagulation cascade, its inhibitors and receptors as specific mediators of either toxic or trophic effects on the nervous system. Our studies utilized *in vitro* tissue culture to probe the effects of thrombin, and inhibitors of thrombin, on neurons and glial cells. Once the thrombin receptor, subsequently named protease-activated receptor 1 (PAR1), was identified and sequenced by Coughlin's group in the early 1990 s, our studies also involved the expression of PAR1 in parts of the nervous system, in particular, the spinal cord as well as in the brain and neuromuscular system. Our translational interest was primarily in SCI and ALS, since the initial studies in neuronal types found exquisite sensitivity of spinal cord motor neurons that lead to apoptotic motor neuronal cell death in culture by thrombin cleavage of the G-protein coupled receptor (GPCR), PAR1. We also explored PARs in AD and PD as well. A number of other groups in Switzerland, Germany, Israel, Italy, Korea, China and Japan, amongst others, as well as the U.S. also began exploring coagulation and fibrinolytic proteases and inhibitors in the nervous system, especially after publication of The Maratea Meeting Proceedings in 1990 (251).

Although the emphasis of this review is on ALS similar results and concepts have been found for other neurodegenerative diseases including AD, PD, and multiple sclerosis (MS) and numerous reviews are available (252). Of interest, until recent evidence for biased signaling (see below) through PAR1 by APC was discovered (253), the previous data indicated that high thrombin concentrations were neurotoxic and pathologic in brain while low thrombin concentrations could induce neuronal and astrocytic survival after various brain insults. Interestingly, thrombin-mediated cell death and cell survival shared initial signaling proteins (48, 254).

## Thrombomodulin (TM) in CNS Development, Neurotrauma, and Neurodegeneration

TM was discovered by Esmon and Owen in the 1970 s and reported in 1982 (255, 256). This discovery came after a decade or more of research that resulted in discovery of Protein C (PC), a vitamin-K dependent factor that is activated by thrombin that results in activated protein C (APC), a serine protease. Initially, the principal role of APC was thought to be its anticoagulant function whereby it proteolytically inactivated FV and FVIII (Figure 1). However, this multi-molecular system, now termed the PC–TM-EPCR (endothelial PC receptor) pathway (257, 258), is a natural mechanism to regulate hemostasis and to integrate it with other host defense system such as innate immunity, inflammation, and to control cell proliferation. Since its cloning, sequencing and chromosomal localization (259), the bulk of studies on TM have also been in terms of its role as a natural anticoagulant. However, as important as this action is, the integration by TM of hemostasis and innate immunity may determine its even greater future in disease processes that affect the CNS.

**Figure 4:**
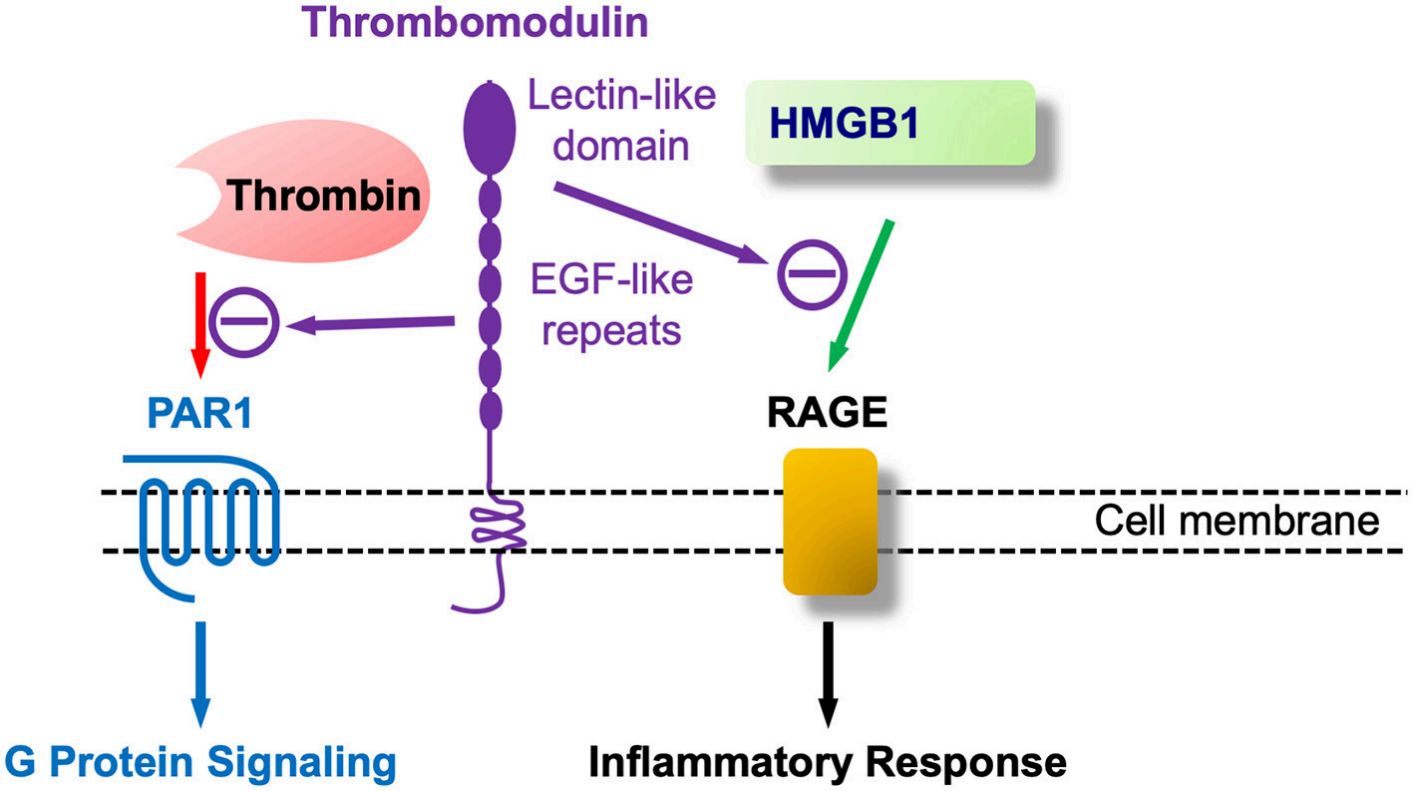
Thrombomodulin (TM) at nexus of coagulation and inflammation (innate immunity): TM as at least a bifunctional molecule that binds coagulation (thrombin) and proinflammatory (HMGB1) agents.

Of interest, shortly after the discovery of TM a report indicated the presence of a surface marker protein in developing mouse parietal endoderm that was modulated by cAMP (260). Shortly thereafter, fetomodulin was found to be identical to TM by contemporary gene cloning techniques (261). Thus, TM or fetomodulin (FM) is present at very early developmental stages and in adults TM expression is greatest in ECs, more predominant in small, microvascular than in large vessel ECs, and was found in essentially all ECs (262). However, the first article concerning TM and CNS vasculature was negative reporting its absence in brain ECs (263). This was not correct since it was subsequently reported that bovine as well-human brain capillaries expressed TM (264, 265), again suggesting its role as a microvascular EC marker. Not surprisingly given its early developmental appearance in parietal endoderm (FM), TM is also expressed in a number of other cells including keratinocytes, osteoblasts, monocytes, neutrophils and chondrocytes, amongst others. We first found that TM was expressed in mouse brain astrocytes, where it was functionally identical to its role in ECs (266). Subsequently, TM was found to be a novel marker of injury-induced astrogliosis, and identified the involvement of thrombin-activated PAR1 (267). This finding suggested its involvement in nervous system injury, i.e., neurotrauma. The TM gene (*THBD*) is intronless and is structurally separated into five distinct domains (see Figure 4). Biochemically, TM is a chondroitin sulfate proteoglycan (CSPG), and consistent with its role as a CNS injury-related CSPG would be increased in the “glial scar” and assemble along with other CSPGs such as neurocan and phosphacan that are also expressed in reactive astrocytes (268).

As shown in Figure 4 TM's additional role in regulating inflammation, apart from coagulation and thrombin's proinflammatory role by PAR1 activation, is largely encoded at its –NH2 terminal, known as the C-type lectin like domain (TM-CTLD). The CTLD is involved in a host of inflammatory diseases, as described in the treatise by Conway (258), one of the leaders in this field. A mechanism for the CTLD in these conditions was provided by the pioneering work of Maruyama's group that discovered that the TM-CTLD bound and neutralized the DAMP alarmin HMGB1 (269). This same group found that HMGB1 was upregulated in spinal cord parenchyma following SCI (270). Beyond its sequestering and neutralization of HMGB1 the TM-CTLD also interferes with complement activation and binds to LPS/endotoxin, and, in Gram-negative bacterial infections, to the Lewis Y antigen (271). Of interest, transgenic mice lacking the NH2-terminal CTLD (TM^LeD/LeD^) have heightened susceptibility to treatment with LPS (258) and should be more vulnerable to weight-drop SCI than wild type mice. The deposition of HMGB1 in the injured spinal cord was shown in rats (270), along with its release into the circulation.

The relationship between the BBB, more precisely the BSCB, and the *coag-inflammation nexus* in ALS merits comment. As mentioned above, although BBB/BSCB dysfunction in ALS was discussed as far back as the 1940 s, it took many decades and newer technology to establish its actual existence. The identification by Garbuzova-Davis and her colleagues that BSCB dysfunction occurred in both ALS patients and fALS SOD1 mice, prior to motor neuron degeneration (220, 221, 226–229), has been confirmed by other groups (222–225, 230, 231). Furthermore, there is a connection between BSCB dysfunction and the PC–TM-EPCR pathway, as shown by the amelioration of motor problems in SOD1 mice by treatment with non-proteolytic/non-anticoagulant APC analogs (224).

A simultaneous activation of the coagulation cascade after injury, as occurs in sepsis, is an ancient host response dating back very early in the evolution of eukaryotes. The contemporary clinical correlate happens in sepsis and injury where excessive thrombin activation develops in disseminated intravascular coagulation (DIC) associated with sepsis and sterile traumatic SIRS (272, 273). A phylogenetic clue into the nexus of clotting and inflammation comes from studies of the omnipresent East Coast North American horseshoe crab, *Limulus polyphemus*, with its open circulatory system containing the hemolymph, and single cell, the amoebocyte, with properties of both platelets and phagocytes. Limulus has survived for >250 million years exposed to LPS or endotoxin in the ocean from *Cyanobacteria* or blue-green algae where they have been for the past 2 billion years (274). The Limulus lysate detection kit for LPS in blood has been in use worldwide for over 30 years. Coagulopathy also commonly develops with TBI since the brain is a rich source of TF and thromboplastin (275).

It should be noted at this point that the thrombin**→**PAR1**→**BBB dysfunction pathogenetic pathway is not specific for ALS but occurs in all situations where intravascular prothrombin activation to α-thrombin exceeds its neutralization either by circulating anti-thrombin (AT) or EC-bound TM and the EPCR (276, 277). This dysfunction pathway would be applicable to AD, PD, ALS and neurodegeneration, in general, especially in those situations associated with antecedent trauma. In this regard, all PARs are expressed on ECs and brain microvascular ECs are no exception. However, PAR1 and PAR4 are also expressed on brain pericytes, which appear to be the most thrombin-sensitive perivascular cells to release membrane metalloprotease-9 (MMP-9) (278, 279). MMP-9 has been shown to cause BBB disruption by proteolyzing tight junction (TJ) proteins (280, 281).

**Figure 5:**
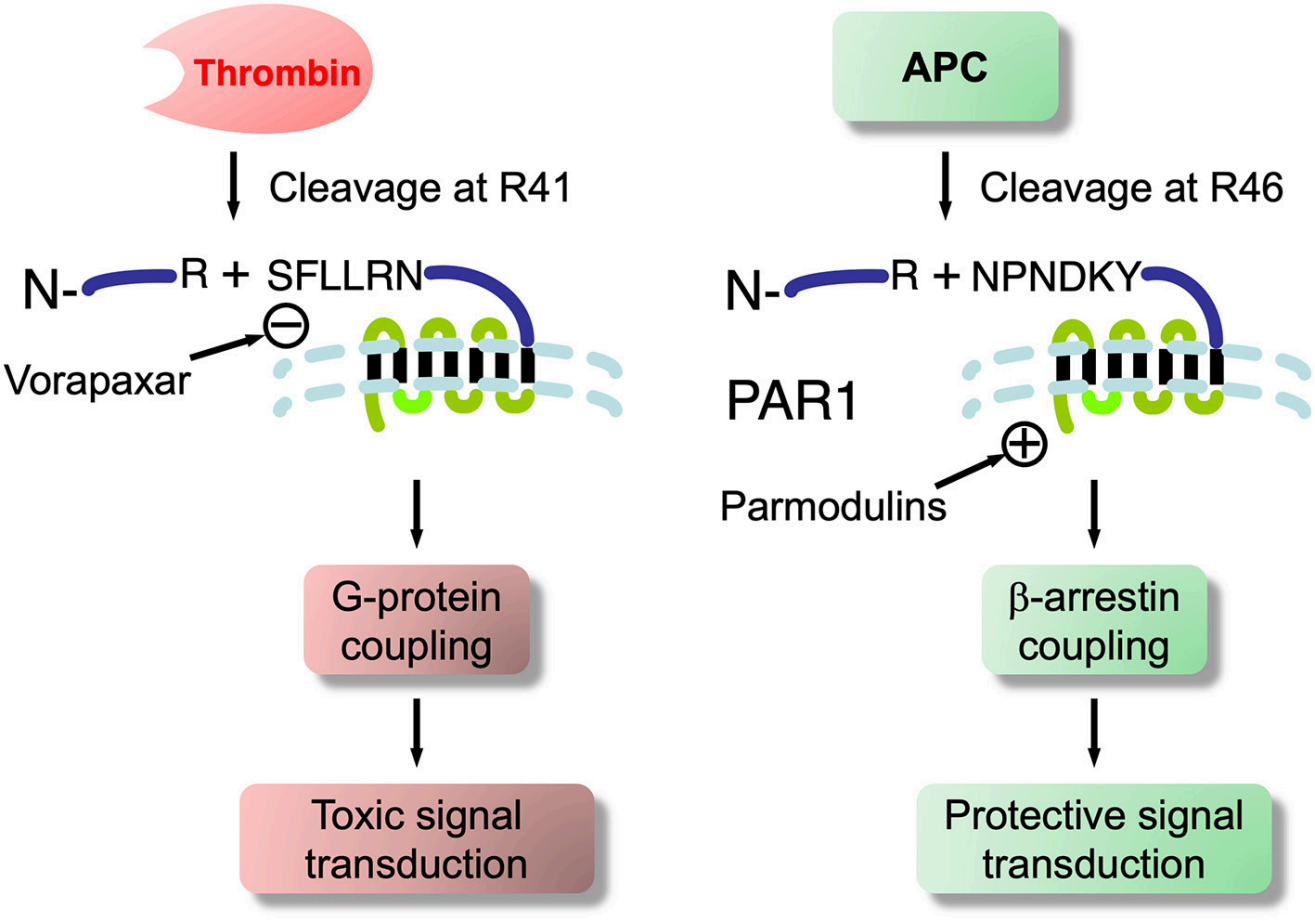
Biased signaling at PAR1 showing thrombin and APC cleavage sites- basis for toxicity and cytoprotection at this G-protein coupled receptor. Also shows synthetic PAR1 antagonist, vorapaxar, an FDA approved therapeutic for cardiovascular conditions. It has been shown to induce endothelial injury, which is associated with BBB/BSCB dysfunction in SOD1 mice and ALS patients. Whether ALS may be triggered in susceptible individuals so treated requires evaluation. Also shown are novel parmodulins that do not have endothelial injury side effects of vorapaxar and, thus, may have therapeutic application to ALS and neurodegenerations.

Recombinant APC (rAPC; drotrecogin alfa, activated; Xigris™) was the first agent shown to stimulate PAR1-mediated cytoprotection approved for human use (in severe sepsis). However, it was voluntarily removed from the market by Eli Lilly in 2011. A number of studies have emphasized the cytoprotective role of APC, encompassing anti-apoptotic and anti-inflammatory activities, as well as significant stabilization of endothelial barriers including the BBB and BSCB. Most studies indicated this was mediated by PAR1 or PAR3 (282). All PARs are expressed on ECs (9, 283, 284) and brain microvascular ECs of the BBB should be no exception. The evidence that thrombin, via PAR1 activation, caused vascular leakage and disruption across various vascular barriers (285–287), including the BBB (288) while APC activation of PAR1 did the reverse, i.e., protection and prevention of leakage (253, 282), was a conundrum. However, as reviewed by Griffin et al. (282) this lead PAR/APC researchers to the notion of “biased signaling”, a phenomenon found in other GPCRs, a group to which PARs belong. Biased signaling through PAR1 for thrombin and APC, as conceived by Griffin et al. is shown in Figure 5: thrombin cleaves PAR1 at ARG41 in the extracellular –NH2 domain, while APC does so at ARG46 (282). At PAR1 thrombin signals through the small GTPase RhoA and ERK1/2 to disrupt, while APC through RAC1, β-arrestin and P13k/Akt to protect. This puts PAR1 on BBB ECs in a very significant position and its different proteolytic ligands to destroy or save BBB function. APC is effective in compression SCI (289) and we found that recombinant TM is also neuroprotective in rat weight-drop contusion SCI (61). More recently, Noble-Haeusslein and colleagues reported that APC biased signaling through PAR1 enhanced locomotor recovery in rat SCI (290). Zlokovic and colleagues showed that treatment with non-proteolytic/non-anticoagulant APC analogs (224) improved motor functions in SOD1 mice.

### PARs in ALS and Other Neurodegenerative Diseases

We found that nM thrombin concentrations induced tau neurofibrillary tangle-like aggregates (NFTs) in murine hippocampal neurons and that this required PAR activation that was followed by delayed synaptophysin reduction and apoptotic neuronal death (291). Subsequently, others showed that the initial fragmentation of tau, necessary to then cause aggregation into NFTs, was due to a thrombin-like protease (292). These authors wrote that fragmentation by a thrombin-like protease was a “prelude” to aggregation, although phosphorylation was not. HIV-associated neurodegeneration (HAND) was also shown to require thrombin and PAR1 expression in astrocytes, as subsequently reviewed (293). McGeer and colleagues then showed that thrombin, as well as prothrombin, accumulated with NFTs in the brains of AD patients (294). Additional evidence for thrombin and PAR1 in neurodegeneration was provided by others in AD (295, 296) and PD (297) and then reviewed as well (50).

Our preliminary data indicated that PARs were increased and active in several murine ALS models in which microglia express increased monocyte chemoattractant protein 1 (MCP-1) and other markers. In regards to neurotrauma, we found that SCI was accompanied by an early and significant upregulation of neurotoxic serine proteases, prothrombin, and PAR1 in the rat spinal cord (298). It was subsequently reported that thrombin-recruited microglia also express MCP-1 (now CCL2) and that PAR1 activation is required for this (299).

The *wobbler* mouse is a model of motor neuron disease sharing many features with ALS, including loss of spinal motor neurons, neuromuscular loss of function over time, and TDP-43 aggregates and C-terminal fragments identical to those seen in the sporadic form of ALS (300). By optimizing transcription and quantitative PCR procedures to facilitate rapid copy number determination in small RNA samples, we documented a 5-fold greater level of PAR1 mRNA in the cervical spinal cord of *wobbler* (wr/wr) compared to wild type mice (301). Then we subsequently confirmed and extended these results showing that PAR1 mRNA was dramatically increased in spinal cord alpha motor neurons in homozygous, spontaneously mutant autosomal recessive *wr/wr* mice (302). The gene for wobbler mutation is located on mouse chromosome 11 (303) and was since shown to be a point mutation on Vps54 (vacuolar protein sorting 54) involved with the Golgi apparatus (304). Even before the gene was determined an informative marker at the wobbler locus, the glutamine synthase 1 (glns-1) pseudogene, permitted genotyping mice prior to phenotype development as previously described (305). Using this technique, we found that homozygotes expressed an 8-fold increase in PAR1 message by P8, more than 2 weeks prior to phenotype detection and this appeared primarily in motor neurons (301–303, 305, 306). These earlier studies focused attention on potential roles of coagulation proteases and PARs in the nervous system but took some time before they had generated additional interest in pursuing direct connections between them, neurotrauma, neuroinflammation and neurodegeneration.

Following our earlier reviews (47, 48, 251, 307) more recent efforts have emphasized the participation of coagulation in various neuroinflammatory diseases of the CNS (64, 308–310). Connections between tissue transglutaminase (tTG), in the same family as the clotting cascade Factor XIII, cross-linking and neuroinflammation in ALS also exist. SCI has been shown to upregulate cytokines, microglia and tTG (311). In addition, we found that SCI induced a “switch” from a GTPase function of tTG to a novel GTP-independent cross-linking isoform in the spinal cord (311). More recently, tTG has been implicated in promoting neuroinflammation in SOD1 mice (312). It would be of interest to determine whether tTG upregulation was present in ALS spinal cord and if alternative transcription to a short isoform existed.

## Epilog, and Possibly, Prolog (to the Next Phase)

Vorapaxar is a natural product-based orthosteric antagonist of thrombin-induced PAR1 that inhibits all signaling downstream (313). The FDA approved it for post-myocardial infarction following success in two large pivotal multi-center Phase III outcome clinical trials in patients with coronary atherothrombosis. It has a low molecular weight (590.7) and a long effective half-life (3–4 days).

Surprisingly, the FDA review of the adverse events for both Phase III clinical trials revealed an increased number of ALS diagnoses in the vorapaxar arm compared to the placebo arm (314). This adverse event was not mentioned in the publication of the results and this vorapaxar-ALS association may recall the studies we and others carried out with thrombin, PAR1, thrombospondin, TM and related components of the *coag-inflamm* system in development, neurotrauma, ALS and other neurodegenerative disorders, as described above (see Table 1). Since vorapaxar appears to inhibit all signaling downstream of the PAR1 GPCR it would seem that is where attention should be paid for clues to ALS pathogenesis related to it. Although it is still a relatively rare occurrence after vorapaxar we would hope that knowledge of this small but surprising ALS *signal* after vorapaxar will uncover novel therapeutic targets for this enigmatic and fatal neurodegenerative disorder and related disorders where synapse retraction may be the earliest pathophysiologic sign of the disease (22, 24–26) and where thrombin**→**PAR1 activation may well play a role (51, 52).

In this regard, recent development of small molecule PAR antagonists termed *parmodulins* (315, 316) are based on findings that biased signaling peptides developed around APC are cytoprotective at PAR1 and not anticoagulant (282). We hope that such research will help advance whether or not potential neuronal degeneration and/or impaired neuromuscular activity is a class-specific adverse effect after PAR antagonists (317).

## Author Contributions

BF conceived of the review, and was lead on the evaluation of the literature and writing. BC also contributed to the reviewing, writing, and figures.

### Conflict of Interest Statement

BF is the Founder of PHLOGISTIX LLC, a startup biotech company. The remaining author declares that the research was conducted in the absence of any commercial or financial relationships that could be construed as a potential conflict of interest.
